# Make Haste Slowly

**Published:** 1874-11

**Authors:** 


### MAKE HASTE SLOWLY.

We are all living too fast. The man who is always in a hurry generally has to do his work over again, besides being more liable to trip up and find himself sprawling in the mud.

We are too fast in our furniture; it is so splendid that our parlors are unused, and are kept so dark, and the chairs and sofas are so completely covered, that, however beautiful they may be, neither family nor friends are ever allowed to enjoy the sight, except on New Year's day, or when a party is given. The best way to enjoy things is to use them, and thus get the worth of our money out of them.

We are too fast in our houses. Sayre, the Kentucky banker, and the greatest good-doer with his money in all the Mississippi Valley, had his "office" in his front parlor, and his family and dining room in the rear. He was always glad to see everybody, especially "preachers." But if one happened to come in when he was making a deep "shave" into a note, he would bow them in to his wife, who was always at hand, always cordial, always chatty, for she "understood things."

The Paris Rothschild for many years had a similar arrangement, saving a million or more francs in rent in the course of a lifetime. Nine-tenths of New York merchants fail in the long run and pass out of sight, because they do not live close themselves, as was the case generally in the recollection of many. An ordinary "store" averages a rent of five thousand dollars; a dwelling, three thousand. Two or three "lofts" of the stores are, in many cases, almost wholly unused, and would give more "house room" than the family needed. Multiply three by twenty years, that being about the "business lifetime" of a merchant, and there is a surplus of sixty thousand dollars on which to live in comfort and dignified retirement for many long years. Who will be the hero among our merchant princes to set the example?

We drive too fast. Going up Fifth Avenue any afternoon, an observant man will be impressed with the number of young men under forty, who are driving their "teams." Nine out of ten of them will, in a score of years, have to walk to the Central Park, if they go there at all.

We stimulate too fast. Passing an eating house near Broadway a young gentleman, perched on a stool before a counter, where as much bread and meat and potatoes, all of the best, as can be eaten are given for a "quarter," was pegging away with all his might at the bottom of a pepper-box, to give "taste" to his food. That young man will, within twenty years, be a confirmed dyspeptic, a drunkard, or an inhabitant of the graveyard. A man under threescore has no business with pepper and mustard and Worcester sauce, for he who uses them drinks too; their employment naturally leads to liquor, and liquor to an early or a dishonored grave. Reader, latinize the heading, and from this good hour let you and yours *Festina Lente.*

